# Phylogenetic and morphological analyses of *Coniochaeta* isolates recovered from Inner Mongolia and Yunnan revealed three new endolichenic fungal species

**DOI:** 10.3897/mycokeys.83.71140

**Published:** 2021-09-09

**Authors:** Hong-Li Si, Yue-Min Su*, Xiao-Xiao Zheng, Meng-Yao Ding, Tanay Bose, Run-Lei Chang

**Affiliations:** 1 College of Life Science, Shandong Normal University, Jinan 250014, Shandong, China Shandong Normal University Jinan China; 2 Department of Biochemistry, Genetics & Microbiology, Forestry and Agricultural Biotechnology Institute (FABI), University of Pretoria, Pretoria 0002, South Africa University of Pretoria Pretoria South Africa

**Keywords:** Coniochaetaceae, lichens, molecular phylogeny, Mongolia, Yunnan Province

## Abstract

Lichens are the result of a symbiotic interaction between fungi (mycobionts) and algae (phycobionts). Aside from mycobionts, lichen thalli can also contain non-lichenised fungal species, such as lichenicolous and endolichenic fungi. For this study, three surveys were conducted in China’s Yunnan Province and Inner Mongolia Autonomous Region between 2017 and 2020. Several samples of four lichen species were collected during these surveys: *Candelariafibrosa*, *Flavoparmeliacaperata*, *Flavopuncteliaflaventior* and *Ramalinasinensis*. Six isolates of *Coniochaeta* were recovered from these four lichen species. The phylogenetic and morphological analyses revealed that two of these isolates were previously identified species, *Coniochaetavelutinosa* and *C.acaciae*. Those remaining were from potentially unknown species. We used molecular and morphological data to describe these previously-unknown species as *Coniochaetafibrosae***sp. nov.**, *C.mongoliae***sp. nov.** and *C.sinensis***sp. nov.** The findings of this study significantly improve our understanding of the variety and habitat preferences of *Coniochaeta* in China and globally.

## Introduction

Lichens are a symbiotic relationship between heterotrophic fungi and algae (including cyanobacteria) that are usually referred to as mycobiont and phycobiont, respectively (Nash and Thomas 2008; Tripathi and Joshi 2019). Lichens exhibit a diversity of colours, thallus morphology and fruiting bodies (Ahmadjian 1993). Lichens have a limited fossil record, yet recent molecular-clock analyses suggested their being at least 250 million years old (Nelsen et al. 2020). Apart from the mycobionts, a lichen thallus can also house non-lichenised fungal species, such as lichenicolous and endolichenic fungi. The former utilise lichens as their hosts (Lawrey and Diederich 2003), whereas the latter behave similar to ‘endophytes’ (Arnold et al. 2009; Suryanarayanan and Thirunavukkarasu 2017). Various species of *Coniochaeta* are examples of endolichenic fungi (Zhang et al. 2016; Harrington et al. 2019).

*Coniochaeta* is a genus of pleomorphic yeasts belonging to the Coniochaetales (Ascomycota) with global distribution (García et al. 2006; Damm et al. 2010; Raja et al. 2012; Vazquez-Campos et al. 2014; Nasr et al. 2018; Harrington et al. 2019). This genus has distinct asexual and sexual states in its life cycle. Previously, the genus *Lecythophora* was erected to include asexual states of *Coniochaeta* (Weber 2002). After the dual nomenclature of pleomorphic fungi was discontinued (Hawksworth 2011), following the principle of priority, these genera were reclassified under *Coniochaeta* (Khan et al. 2013; Réblová et al. 2016).

The sexual state of *Coniochaeta* is characterised by dark brown to black ascomata with setae. These ascomata can either be pyriform ostiolate or globose non-ostiolate. Asci are thin-walled, producing single-celled, smooth ascospores with an elongated embryo crack (García et al. 2006; Asgari et al. 2007). In contrast, the asexual state of *Coniochaeta* has distinctive pink salmon to dark brown colonies producing phialidic conidiogenous cells (Checa 1988; Damm et al. 2010; Khan et al. 2013). *Coniochaeta* has been isolated from various substrates, such as butter, faeces, wood, soil, uranium wastewater, plants and lichens (Weber 2002; García et al. 2006; Vazquez-Campos et al. 2014; Harrington et al. 2019). Some *Coniochaeta* species are also known to be human and animal pathogens (Hoog et al. 2000; Perdomo et al. 2013; Troy et al. 2013).

Several *Coniochaeta* species have been isolated from Asia (Kamiya et al. 1995; García et al. 2006; Asgari et al. 2007). Previously, three undescribed *Coniochaeta* species were identified from China growing on plant litters and herbivore faeces, but none associated with liches (Chang and Wang 2011; Hyde et al. 2020). In this study, six isolates of *Coniochaeta* species were recovered from four lichen species collected from the Yunnan Province and the Inner Mongolia Autonomous Region of China. Analyses of molecular and morphological data indicated these six isolates represented five species of *Coniochaeta*. Amongst these were two previously-described taxa, *C.velutinosa* and *C.acaciae*, whereas the remaining three were undescribed. Here, we describe these species as *Coniochaetamongoliae* sp. nov., *C.sinensis* sp. nov. and *C.fibrosae* sp. nov. This study substantially augments our current knowledge on the diversity and host range of *Coniochaeta* and endolichenic fungi from China.

## Materials and methods

### Collection of lichen samples

Between 2017 and 2020, three surveys were conducted in the Yunnan Province and Inner Mongolia Autonomous Region of China. During these surveys, multiple samples of four lichens species were collected. Samples of *Flavoparmeliacaperata* (2017), *Flavopuncteliaflaventior* (2017) and *Candelariafibrosa* (2020) were collected from the Yunnan Province, whereas *Ramalinasinensis* was collected from the Inner Mongolia Autonomous Region in 2019. During their transit, all lichen samples were stored separately in paper bags.

### Isolation of fungi from lichen thalli

All lichen samples were repeatedly rinsed with tap water followed by deionised water. Using a Leica Zoom 2000 stereomicroscope, the upper cortex was scraped off with a sterile blade. The medullary layer was carefully dissected and rinsed using sterile deionised water. Thereafter, these medullary tissues were placed on to 2% potato dextrose agar (PDA) plates, amended with 0.05% streptomycin. All Petri plates were incubated for 14 days at 25 °C. Hyphal tips of mycelia emerging from the medullary tissues were sub-cultured on to fresh PDA plates.

Ex-holotype cultures of undescribed fungal species, described in this study, were deposited in the China General Microbiological Culture Collection Center (CGMCC), Beijing, China. The holotype specimens were deposited in the culture collection of the Institute of Microbiology (HMAS), Beijing, China (Accession numbers are listed in Table. 1).

**Table 1.** GenBank accession numbers *Coniochaeta* species used for the phylogenetic analyses. T = ex-type isolates.

Taxa	Strain	HMAS	GenBank accession number		
			LSU	ITS	
* Coniochaeta acaciae *	MFLUCC 17-2298^T^		MG062737	MG062735	
*** C. acaciae ***	**CX37**		**MW750757**	**MW750761**	
* C. africana *	CBS:120868^T^		NG_066150	NR_137725	
* C. angustispora *	CBS:144.70		MH871308	MH859528	
* C. arenariae *	MFLUCC 18-0405^T^		MN017893	-	
* C. baysunika *	MFLUCC 17-0830^T^		MG828996	MG828880	
* C. boothii *	CBS:381.74^T^		AJ875226	NR_159776	
* C. cateniformis *	UTHSC 01-1644^T^		HE610329	NR_111517	
* C. cephalothecoides *	L821		KY064030	KY064029	
* C. coluteae *	MFLUCC 17-2299^T^		MG137252	MG137251	
* C. cruciata *	FMR 7409		AJ875222	-	
* C. cymbiformispora *	NBRC 32199		LC146726	LC146726	
* C. cipronana *	CBS:144016^T^		-	NR_157478	
* C. decumbens *	CBS:153.42^T^		NG_067257	NR_144912	
* C. dendrobiicola *	DLCCR7		MK225603	MK225602	
* C. discoidea *	CBS:158.80^T^		NG_064120	NR_159779	
* C. discospora *	CBS:168.58		MH869278	MH857740	
* C. ellipsoidea *	CBS:137.68^T^		MH870804	MH859091	
* C. endophytica *	AEA 9094^T^		EF420069	EF420005	
* C. euphorbiae *	CBS:139768 = 1001^T^		-	KP941076	
* C. extramundana *	CBS:247.77^T^		MH872828	MH861057	
* C. fasciculata *	CBS:205.38^T^		FR691988	NR_154770	
*** C. fibrosae ***	**CGMCC3.20304^T^**	**350271**	**MW750758**	**MW750760**	
*** C. fibrosae ***	**CX04D1**		**MW750755**	**MW750756**	
* C. fodinicola *	FRL = CBS:136963^T^		KF857172	JQ904603	
* C. gigantospora *	ILLS:60816^T^		JN684909	JN684909	
* C. hansenii *	CBS:885.68		AJ875223	-	
* C. hoffmannii *	CBS:245.38^T^		AF353599	NR_167688	
* C. iranica *	CBS:139767 = 0806^T^		-	KP941078	
* C. krabiensis *	MFLU 16-1230^T^		MN017892		-
* C. leucoplaca *	CBS:486.73		MH872465		-
* C. ligniaria *	98.1105		AF353585		-
* C. lignicola *	CBS:267.33^T^		NG_067344		NR_111520
* C. luteorubra *	UTHSC 01-20^T^		HE610328		HE610330
* C. luteoviridis *	CBS:206.38^T^		NG_067348		NR_154769
* C. malacotricha *	F2106		AF353589		-
* C. marina *	MFLUCC 18-0408^T^		MK458765		MK458764
* C. mutabilis *	CBS:157.44^T^		NG_042382		NR_111519
* C. navarrae *	LTA3 = CBS:141016^T^		KU762326		KU762326
* C. nepalica *	NBRC 30584^T^		LC146727		LC146727
* C. ornata *	FMR7415^T^		AJ875228		-
* C. ostrea *	CBS:507.70^T^		NG_064080		NR_159772
* C. polymorpha *	CBS:132722^T^		HE863327		NR_121473
* C. polysperma *	CBS:669.77^T^		MH872868		MH861109
* C. prunicola *	CBS:120875^T^		GQ154602		GQ154540
* C. pulveracea *	CAB683		GQ351559		-
* C. punctulata *	CBS:159.80		MH873024		MH861254
*** C. mongoliae ***	**CGMCC3.20250^T^**	**350270**	**MW077646**		**MW077645**
* C. rhopalochaeta *	CBS:109872^T^		GQ351561		-
* C. rosae *	TASM:6127^T^		NG_066204		NR_157509
* C. savoryi *	CBS:725.74^T^		MH872627		MH860890
* C. simbalensis *	NFCCI:4236^T^		MG917738		NR_164024
*** C. sinensis ***	**CGMCC3.20306^T^**	**350269**	**MW422265**		**MW422269**
* C. sordaria *	CBS:492.73		MH878380		-
* C. subcorticalis *	CBS:551.75		AF353593		-
* C. taeniospora *	LTA = CBS:141014^T^		KU762324		KU762324
* C. tetraspora *	CBS:139.68		MH870806		MH859093
* C. velutina *	CBS:981.68		MH870991		MH859264
* C. velutinosa *	Co29		GU553330		GU553327
*** C. velutinosa ***	**CGMCC3.20249**		**MW346687**		**MW298866**
* C. verticillata *	CBS:816.71^T^		AJ875232		NR_159774
* C. vineae *	KUMCC 17-0322^T^		-		NR_168225
* C. canina *	UTHSC 11-2460		NG_042720		NR_120211
* Zanclospora jonesii *	MFLUCC15-1015^T^		NG_067549		KY212753
* Paragaeumannomyces garethjonesii *	MFLUCC 15-1012^T^		NG_059017		KY212751

### Morphology and growth studies

Colony morphologies of ex-holotypes, representing four potentially new fungal species, were described from eight-day-old cultures growing at 25 °C. A Leica DM6 compound microscope attached to a Zeiss Axio Imager Z2 camera was used for measuring and photographing microscopic morphological characters. A minimum of 50 conidia and conidiogenous cells per isolate were measured using the software ImageJ (Rasband 1997; Schneider et al. 2012).

For the growth study, ex-holotype isolates were sub-cultured on to PDA and incubated for five days at 25 °C. Thereafter, 5 mm diam. agar plugs were placed at the centre of 90 mm Petri dishes. Three replicates per ex-type isolate were incubated at 5, 10, 15, 20, 25, 30 and 35 °C (± 0.5 °C). The colony diameter of each isolate was measured daily up to the eighth day.

### DNA extraction, PCR amplification and sequencing

For all undescribed fungal species, eight-day-old cultures growing at 25 °C were used for the extraction of total genomic DNA using PrepMan^TM^ Ultra Sample Preparation Reagent (Applied Biosystems, California, USA), following the manufacturer’s instructions. The complete internal transcribed spacers (ITS) and the partial 28S nuclear ribosomal large subunit rRNA gene (LSU) were amplified using the primer pairs ITS1/ITS4 (White et al. 1990) and LR0R/LR5 (Vilgalys and Hester 1990; White et al. 1990), respectively.

Each 25 μl of PCR reaction included 10.5 μl of PCR grade water, 12.5 μl of 1–5^TM^ 2× High-Fidelity Master Mix (buffer, MgCl_2_, dNTPs and Taq; Tsingke Co., China), 0.5 μl each of forward and reverse primers and 1 μl DNA template. For both gene regions, PCR amplifications were conducted with an initial denaturation at 94 °C for 3 min, followed by 30 cycles of 94 °C for 30 sec, 56 °C for 1 min, 72 °C for 1 min; final extension at 72 °C for 10 min. Positive amplifications were verified using agarose gel electrophoresis.

All the PCR products were sequenced by QingDao MDBio Biotech Co., Ltd., China. The resulting sequences were assembled using Geneious v.10.2.2 (Biomatters, Auckland, New Zealand). Preliminary identification of the sequences was undertaken using the BLAST algorithm (Altschul et al. 1990) available through the NCBI GenBank. All the sequences, generated in this study, were deposited at GenBank (Table 1).

### Phylogenetic analyses

For the purpose of phylogenetic analyses, we constructed three separate datasets. These are as follows: a) ITS, b) LSU and c) ITS + LSU. Each dataset included sequences generated in this study and those retrieved from the NCBI GenBank. Where available, ex-type sequences of previously-known *Coniochaeta* species were added to the datasets. For all three datasets, *Paragaeumannomycesgarethjonesii* and *Zanclosporajonesii* were selected as the outgroup taxa (Table 1). All datasets were aligned using MAFFT v. 7 (Katoh and Standley 2013); thereafter, manually adjusted if needed using MEGA v.7 (Kumar et al. 2016). All aligned sequence datasets were deposited to TreeBase (Acc. No 28404).

Software for Maximum Likelihood (ML) and Bayesian Inference (BI) phylogenetic analysis was accessed through the CIPRES Science Gateway platform (Miller et al. 2010). jModeltest 2.2 (Nylander et al. 2008) was used for selecting appropriate substitution models. ML analyses were done using RAxML v. 8.2.4 (Stamatakis 2006; Stamatakis et al. 2008) using the GTR substitution model and 1000 bootstrap replicates. BI analyses were undertaken using MrBayes v.3.2 (Ronquist et al. 2012). Four MCMC chains were run from a random starting tree for five million generations and trees were sampled every 100^th^ generation. A quarter of the sampled trees were discarded during burn-in. The remaining trees were used for constructing consensus trees. The resulting ML and BI trees were viewed with FigTree v.1.4 (Rambaut 2009).

## Results

### Isolation

In this study, four lichen species were collected from Yunnan Province and the Inner Mongolia Autonomous Region in 2017, 2019 and 2020. A total of six isolates of *Coniochaeta* were recovered from these four lichen species. These are CX03C1 and CX04D1 from *Candelariafibrosa*, 8004b from *Flavoparmeliacaperata*, CS-04 and CS-09 from *Ramalinasinensis* and CX37 from *Flavopuncteliaflaventior*.

Preliminary identification of these isolates, using the BLAST algorithm, indicated isolates 8004b and CX37 were known *Coniochaeta* species, *C.velutinosa* and *C.acaciae*, respectively, whereas, CX03C1, CX04D1, CS-04 and CS-09 were potentially undescribed species.

### Phylogenetic analyses

**Figure 1.**
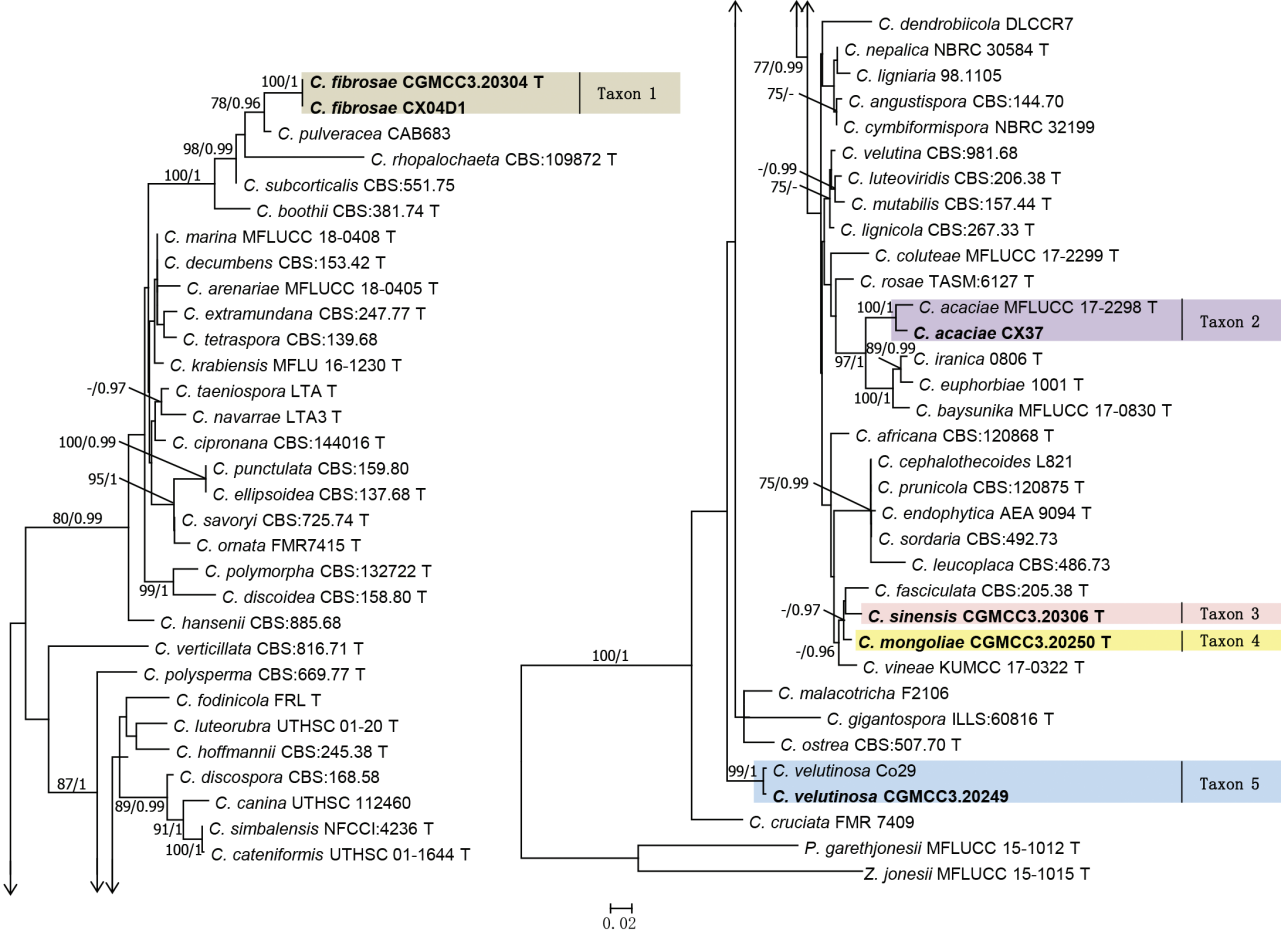
Maximum Likelihood tree constructed using ITS+LSU dataset. Bootstrap support values ≥ 75% and posterior probabilities ≥ 0.95 are indicated above the nodes as ML / PP. The isolates obtained in this study are shown in bold. T = ex-type isolates.

Both single gene and concatenated datasets were used for phylogenetic analyses using ML and BI approaches. The single gene dataset for ITS included 53 taxa, whereas the LSU had 61 taxa. The concatenated dataset included 65 taxa and 1489 characters including gaps (ITS: 1–655; LSU: 656–1489). Individual gene trees for *Coniochaeta* species had similar topologies and were congruent with the tree generated using the concatenated dataset when taxon sampling overlapped. Bootstrap values < 75% and posterior probability < 0.95 were considered unreliable (Fig. 1, Suppl. material 1 and Suppl. material 2).

In the phylogenetic trees, constructed using the concatenated dataset, isolates CX03C1 and CX04D1 formed a monophyletic clade (Taxon 1) and sister to *C.pulveracea* (Fig. 1). Even though, in the phylogenetic trees using a single gene, isolates of Taxon 1 emerged as a monophyletic clade, yet the sister taxon varied. For ITS, *C.boothii* was found sister to Taxon 1, whereas for LSU, it was *C.pulveracea* (Suppl. material 1: Fig. S1 and Suppl. material 2: S2).

In the tree constructed using the concatenated dataset, isolate CX37 (Taxon 2) formed a monophyletic clade with *C.acaciae* with high statistical support. Similar topologies were also observed in the ITS and LSU trees.

The phylogenetic position of isolates CS-04 (Taxon 3) and CS-09 (Taxon 4) substantially varied across the phylogenetic trees. In the trees using the concatenated dataset, isolates CS-04 (Taxon 3) and CS-09 (Taxon 4) nested within a clade that included *C.fasciculata* and *C.vineae* (Fig. 1). In ITS gene trees, isolates CS-04 and CS-09 nested within a clade that included *C.coluteae*, *C.fasciculata* and *C.vineae* (Suppl. material 1). In the LSU trees, isolate CS-04 (Taxon 3) grouped with a clade that included *C.leucoplaca*, *C.cephalothecoides*, *C.endophytica*, *C.prunicola* and *C.sordaria* (Suppl. material 2), whereas, isolate CS-09 formed a monophyletic clade with *C.mutabilis* (Suppl. material 2). Irrespective of the trees, the statistical support for all the groups was unreliable.

Irrespective of the datasets and phylogenetic approaches, isolate 8004b (Taxon 5) was grouped with *C.velutinosa* (Asgari and Zare 2006) with high statistical support (Fig. 1, Suppl. material 1 and Suppl. material 2).

### Taxonomy

#### Coniochaeta fibrosae

Taxon classificationFungiConiochaetalesConiochaetaceae

H. L. Si & Y. M. Su sp. nov.

6DC014B1-5414-520D-B337-698C4393AC2C

839390

Figure 2

##### Holotype.

China, Yunnan Province: Tiesuo township, 26°32'71"N, 100°57'3"E, ca. 2120 m elev., isolated from *Candelariafibrosa*, 13 Nov 2020, H. L. Si, CX03C1 (HMAS 350271, holotype), ex-type culture CGMCC3.20304.

##### Etymology.

The name relates to the lichen *Candelariafibrosa* and both isolates of this fungus were isolated from its medulla.

##### Description.

**Figure 2.**
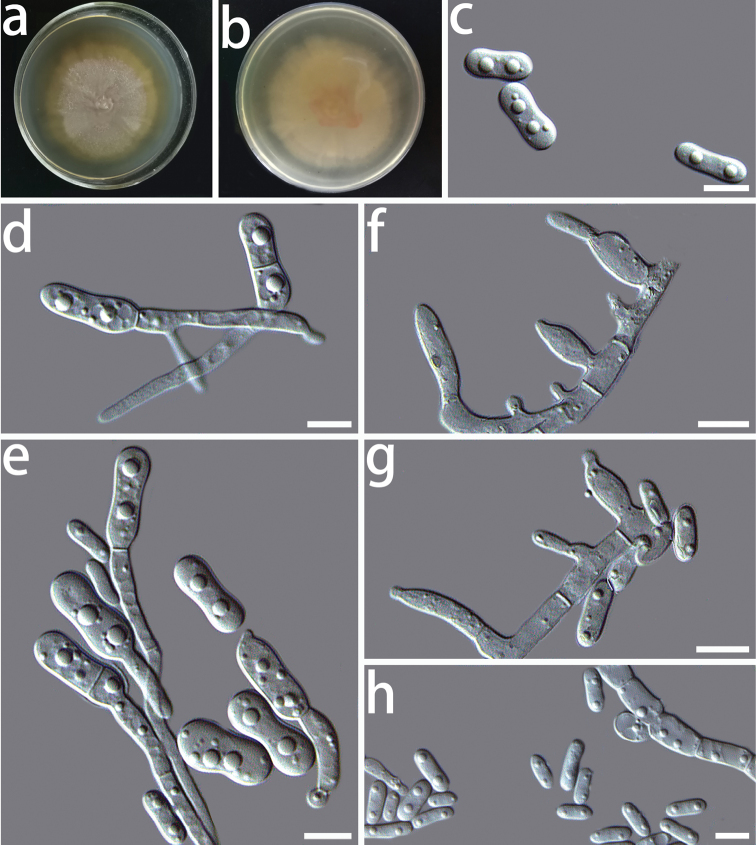
Morphological characters of *Coniochaetafibrosae* sp. nov. (HMAS 350271) **a, b** cultures on PDA from the surface and reverse **c** swollen conidia **d, e** swollen conidia germinate hyphae **f, g** conidiogenous cells **h** conidia. Scale bars: 10 μm.

Colony on PDA after 8 d, hyphae hyaline, multi-guttulate, septate, smooth-walled; conidiophores short; conidiogenous cells hyaline, phialidic or oval in shape, single or in clusters on short lateral branches, measuring 2.9–7.2 × 1.8–3.7 μm (x̄= 4.7 × 2.6 μm, n = 50) (Fig. 2f, g); two types of conidia were observed, swollen conidia were hyaline, one-celled, dumb-bell-shaped, with hyphae emerging from both ends (Fig. 2d, e), measuring 7.6–16.5 × 2.3–4.1 μm (x̄ = 9.7 × 3.1 μm, n = 50) (Fig. 2c), oblong conidia were hyaline, one-celled, often oblong to ellipsoidal in shape, measuring 3.4–6.8 × 1.4–2.7 μm (x̄ = 4.7 × 1.8 μm, n = 50) (Fig. 2h). Chlamydospores absent. Sexual morph unknown.

##### Culture characteristics.

The optimal temperature for growth was 25 °C on PDA. No growth was detected at 5 and 35 °C. Colonies on PDA after 8 d at 25 °C were white, circular, margin entire, flat, dense, partially immersed in the medium and sticky protuberance at the centre of the colony.

##### Additional specimen examined.

China, Yunnan Province: Tiesuo township, 26°32'71"N, 100°57'3"E, ca. 2120 m elev., isolated from on *Candelariafibrosa*, 13 Nov 2020, H. L. Si, CX04D1.

##### Notes.

In the phylogenetic analyses, both isolates of *C.fibrosae* sp. nov. formed a monophyletic clade, but the sister taxon differed between datasets. These sibling species were either *C.boothii* (ITS) or *C.pulveracea* (LSU and concatenated). Both of these sibling species were described, based on their sexual state and chlamydospores (Manoharachary and Ramarao 1973; Romero et al. 1999; García et al. 2006). However, we did not find sexual reproductive structures in our species. As a result, we were unable to compare the morphology of these species.

#### Coniochaeta sinensis

Taxon classificationFungiConiochaetalesConiochaetaceae

H. L. Si & Y. M. Su sp. nov.

4E57B146-2AA6-53BF-8D09-25FAD27BF614

839388

Figure 3

##### Holotype.

China, the Inner Mongolia Autonomous Region: Chifeng City, 44°13'46"N, 118°44'57"E, ca. 1500 m elev., isolated from the medulla of *Ramalinasinensis*, 11 Oct 2019, H. L. Si, CS-04 (HMAS 350269, holotype), ex-type culture CGMCC3.20306.

##### Etymology.

The name relates to the lichen *Ramalinasinensis*, as a single isolate of this fungus was obtained from the medulla of this lichen.

##### Description.

**Figure 3.**
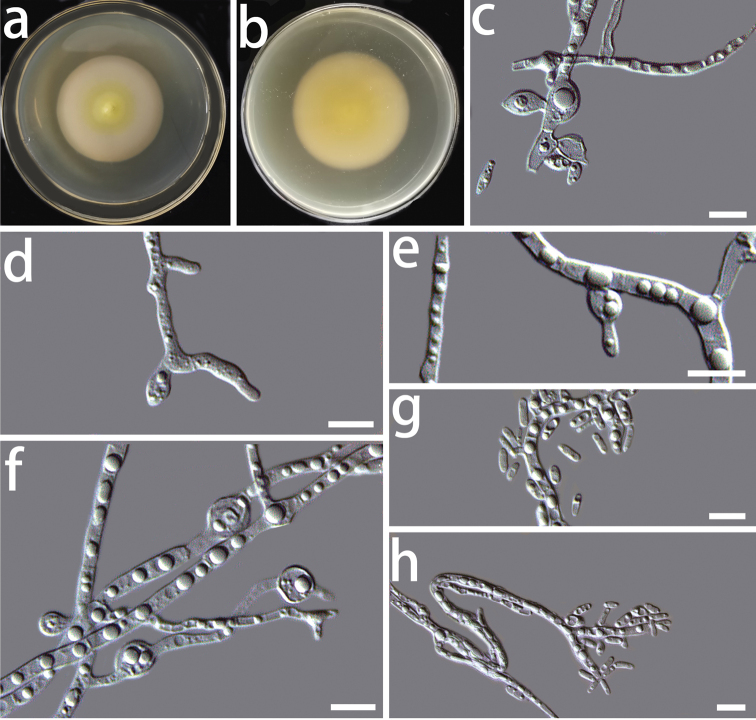
Morphological characters of *Coniochaetasinensis* sp. nov. (HMAS 350269) **a, b** cultures on PDA from the surface and reverse **c, d** conidiogenous cells **e** conidiogenous cell that is producing conidia **f** chlamydospores **g, h** conidia. Scale bars: 10 μm.

Colony on PDA after 8 d, hyphae hyaline, multi-guttulate, septate, smooth-walled, often hyphal strands consolidating to form bundles, conidiophores short or absent; conidiogenous cells hyaline, phialidic or oval in shape, single or in clusters on short lateral branches, measuring 2.8–7.1 × 1.1–3.7 μm (x̄ = 4.2 × 2.3 μm, n = 50) (Fig. 3c–e); conidia hyaline, one-celled, often oblong to ellipsoidal in shape, measuring 2.5–4.6 × 0.7–2.1 μm (x̄ = 3.3 × 1.2 μm, n = 50) (Fig. 3g, h); chlamydospore solitary or in short chains, hyaline, thick-walled, elongate ellipsoidal or almost globose in shape, measuring 3.7–6.6 × 2.5–5.4 μm (x̄ = 4.8 × 3.7 μm, n = 50) (Fig. 3f). Sexual morph unknown.

**Figure 4.**
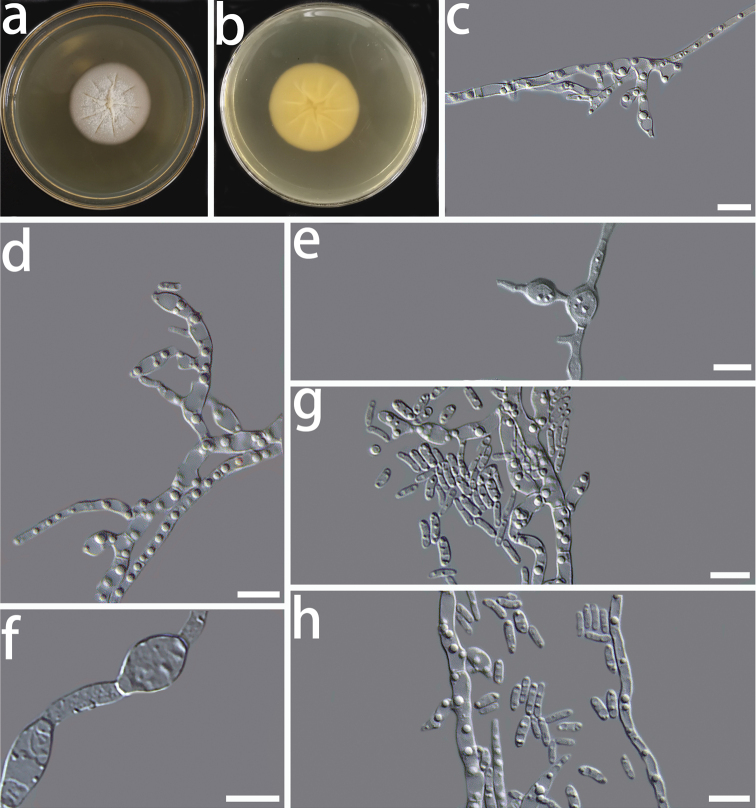
Morphological characters of *Coniochaetamongoliae* sp. nov. (HMAS 350270) **a, b** cultures on PDA from the surface and reverse, **c, d** conidiogenous cells **e, f** chlamydospores **g, h** conidia. Scale bars: 10 μm.

##### Culture characteristics.

The optimal temperature for growth is 30 °C. No growth was detected at 5 and 35 °C. Colonies on PDA after 8 d at 30 °C were yellow in the centre and white around the edges, circular, margin entire, flat, dense, partially immersed in the medium, the centre of the colony slightly bulging.

##### Notes.

*Coniochaetasinensis* sp. nov. clusters with *C.vineae*, *C.fasciculata* and *C.mongoliae* sp. nov. in our phylogenetic tree, constructed using the concatenated dataset, but the statistical support was insignificant. Amongst these species, *C.vineae* is only known in its sexual morph (Hyde et al. 2020). There are, however, significant morphological differences amongst *C.sinensis* sp. nov., *C.fasciculata* and *C.mongoliae* sp. nov. These are (1) the shapes and sizes of conidiogenous cells, (2) the shapes and sizes of conidia and (3) the shapes and sizes of chlamydospores. When compared to *C.mongoliae* sp. nov., *C.sinensis* sp. nov. has smaller conidiogenous cells and conidia. The conidia of *C.sinensis* sp. nov. are significantly smaller than those of *C.fasciculata* (Beyma 1939). Aside from that, the chlamydospores of *C.sinensis* sp. nov. are longer than those of *C.mongoliae* sp. nov.

#### Coniochaeta mongoliae

Taxon classificationFungiConiochaetalesConiochaetaceae

H. L. Si & Y. M. Su sp. nov.

F4350B40-E623-595F-9E93-A90FC25D35DD

839389

Figure 4

##### Holotype.

China, the Inner Mongolia Autonomous Region, Chifeng City, 44°13'46"N, 118°44'57"E, ca. 1500 m elev., isolated from the medulla of *Ramalinasinensis*, 11 Oct 2019, H. L. Si, CS-09 (HMAS 350270, holotype), ex-type living culture, CGMCC 3.20250.

##### Etymology.

The lichen was collected in the Inner Mongolia Autonomous Region, thus the name.

##### Description.

Colony on PDA after 8 d, hyphae hyaline, multi-guttulate, septate, smooth-walled, often with hyphal strands consolidating to form bundles; conidiophores short or absent; conidiogenous cells hyaline, flask or acicular in shape, measuring 3.3–12.5 × 1.6–5.1 μm (x̅ = 6.6 × 2.9 μm, n = 50) (Fig. 4c, d); conidia hyaline, smooth-walled, ellipsoidal, 3.3–8.4 × 0.6–1.9 μm (x̅ = 4.8 × 1.3 μm, n = 50) (Fig. 4g and h); chlamydospore solitary or in short chains, hyaline, thick-walled, elongate ellipsoidal or almost globose in shape, measuring 2.7–6.7 × 2.6–5.4 μm (x̅ = 4.6 × 3.8 μm, n = 50) (Fig. 4e, f). Sexual morph unknown.

##### Culture characteristics.

The optimal temperature for growth is 25 °C. No growth was detected at 5 °C and 35 °C. Colonies on PDA after 8 d at 25 °C were white to light pink in colour, circular, flat, dense, partially immersed in the medium, the centre of the colony is rough, forming radial grooves.

##### Notes.

In the phylogenetic tree using the concatenated dataset, *Coniochaetamongoliae* sp. nov. clustered in a clade that included *C.sinensis* sp. nov., *C.vineae* and *C.fasciculata*, but with low statistical support. Moreover, these four species have substantial morphological differences (for details, see the notes for *C.sinensis* sp. nov.).

## Discussion

In the present study, *Candelariafibrosa*, *Flavoparmeliacaperata*, *Flavopuncteliaflaventior* and *Ramalinasinensis* were collected from the Yunnan and Inner Mongolia Regions of China between 2017 and 2020. We isolated six *Coniochaeta* isolates from these lichens, which we classified into five species. Two of these were previously-described species, while the other three were unknown. Here, we describe these three previously-unknown species as *C.fibrosae* sp. nov., *C.sinensis* sp. nov. and *C.mongoliae* sp. nov.

The majority of species in the genus *Coniochaeta* are saprophytes or pathogens of plants and humans, while many others have an unknown ecological function (Harrington et al. 2019). Species of *Coniochaeta* are frequently isolated from asymptomatic tissues of woody plants and lichens throughout temperate and northern North America (Del Olmo-Ruiz 2012). Some of these species were found exclusively on plants or lichens, such as *C.endophytica* and *C.hoffmannii*, respectively (Zhang et al. 2016; Harrington et al. 2019) or on both, such as *Coniochaeta* sp. Clade 9 (Del Olmo-Ruiz 2012). The two previously-described species recovered in this study (*C.acaciae* and *C.velutinosa*) were also isolated from barley leaves in Iran (Asgari and Zare 2006) and dead *Acacia* species branches in Uzbekistan (Samarakoon et al. 2018). This demonstrates *Coniochaeta*’s ability to thrive in a variety of habitats, yet their ecological role in all these environments is still largely unknown.

The lack of sequences for protein-coding gene regions is one of the pitfalls in identifying taxa in the genus *Coniochaeta*. For the majority of species, only ITS and LSU sequences are currently available. Sequences for the largest subunit of RNA polymerase II (rpb1), the second-largest subunit of RNA polymerase II (rpb2), translation elongation factor 1-alpha (tef1) or β-tubulin gene (tub2) were only used in a few studies involving a limited number of species (Spatafora et al. 2006; Voglmayr et al. 2016; Samarakoon et al. 2018; Harrington et al. 2019). Due to the paucity of sequences, we could not include those gene regions in this study. Moreover, even after repeated attempts, we could not amplify the rpb1, rpb2 and tub2 gene regions for the species isolated in this study. Consequently, there is an urgent need for primers that can successfully amplify protein-coding genes from a wide variety of taxa in order to demystify the taxonomy for this genus.

In this study, we identified the isolate CX37 as *Coniochaetaacaciae*. This is because, in the phylogenies using both concatenated and single-gene datasets, isolate CX37 and ex-type sequences of *C.acaciae* grouped into a monophyletic clade. However, pair-wise comparison of gene regions showed there were at least 15 bps (ITS) and 6 bps (LSU) differences between CX37 and ex-type sequences of *C.acaciae* (Samarakoon et al. 2018). Moreover, following the protocol suggested by Damm et al. (2010) and Harrington et al. (2019), we could not induce ascomata formation in the isolate CX37. This hindered us from comparing the sexual structures of this species. In the future, the discovery of more isolates of *C.acaciae* will allow us to clarify the taxonomy of this species.

In the present study, through repeated sampling, we isolated five *Coniochaeta* species associated with four lichen species in China. Amongst these, three were previously-undescribed species. Data emerging from this study substantially augmented our current knowledge on the diversity and host range of this genus in China and globally. However, our surveys were exclusively conducted in two Provinces in China. Currently, more surveys should be conducted in various ecoregions of China to catalogue the diversity of *Coniochaeta* and various other endolichenic fungi.

## Supplementary Material

XML Treatment for Coniochaeta fibrosae

XML Treatment for Coniochaeta sinensis

XML Treatment for Coniochaeta mongoliae

## Supplementary materials

Supplementary material 1 Figure S1. ML tree generated from ITS sequence data Data type Pdf. file Explanation note Maximum Likelihood tree constructed using ITS dataset. Bootstrap support values ≥ 75% and posterior probabilities ≥ 0.95 are indicated above the nodes as ML / PP. The isolates obtained in this study are shown in bold. T = ex-type isolates. https://binary.pensoft.net/file/586584This dataset is made available under the Open Database License (http://opendatacommons.org/licenses/odbl/1.0/). The Open Database License (ODbL) is a license agreement intended to allow users to freely share, modify, and use this Dataset while maintaining this same freedom for others, provided that the original source and author(s) are credited.Hong-Li Si, Yue-Min Su, Xiao-Xiao Zheng, Meng-Yao Ding, Tanay Bose, Run-Lei Chang

Supplementary material 2 Figure S2. ML tree generated from LSU sequence data Data type Pdf. file Explanation note Maximum Likelihood tree constructed using LSU dataset. Bootstrap support values ≥ 75% and posterior probabilities ≥ 0.95 are indicated above the nodes as ML / PP. The isolates obtained in this study are shown in bold. T = ex-type isolates. https://binary.pensoft.net/file/586585This dataset is made available under the Open Database License (http://opendatacommons.org/licenses/odbl/1.0/). The Open Database License (ODbL) is a license agreement intended to allow users to freely share, modify, and use this Dataset while maintaining this same freedom for others, provided that the original source and author(s) are credited.Hong-Li Si, Yue-Min Su, Xiao-Xiao Zheng, Meng-Yao Ding, Tanay Bose, Run-Lei Chang
