# Supplementary figures and images for: Phylogenetic and morphological analyses of Coniochaeta isolates recovered from Inner Mongolia and Yunnan revealed three new endolichenic fungal species
Source: MycoKeys. 2021 Sep 9;83:105–21. doi: 10.3897/mycokeys.83.71140 (PMC8445908; doi:10.3897/mycokeys.83.71140)

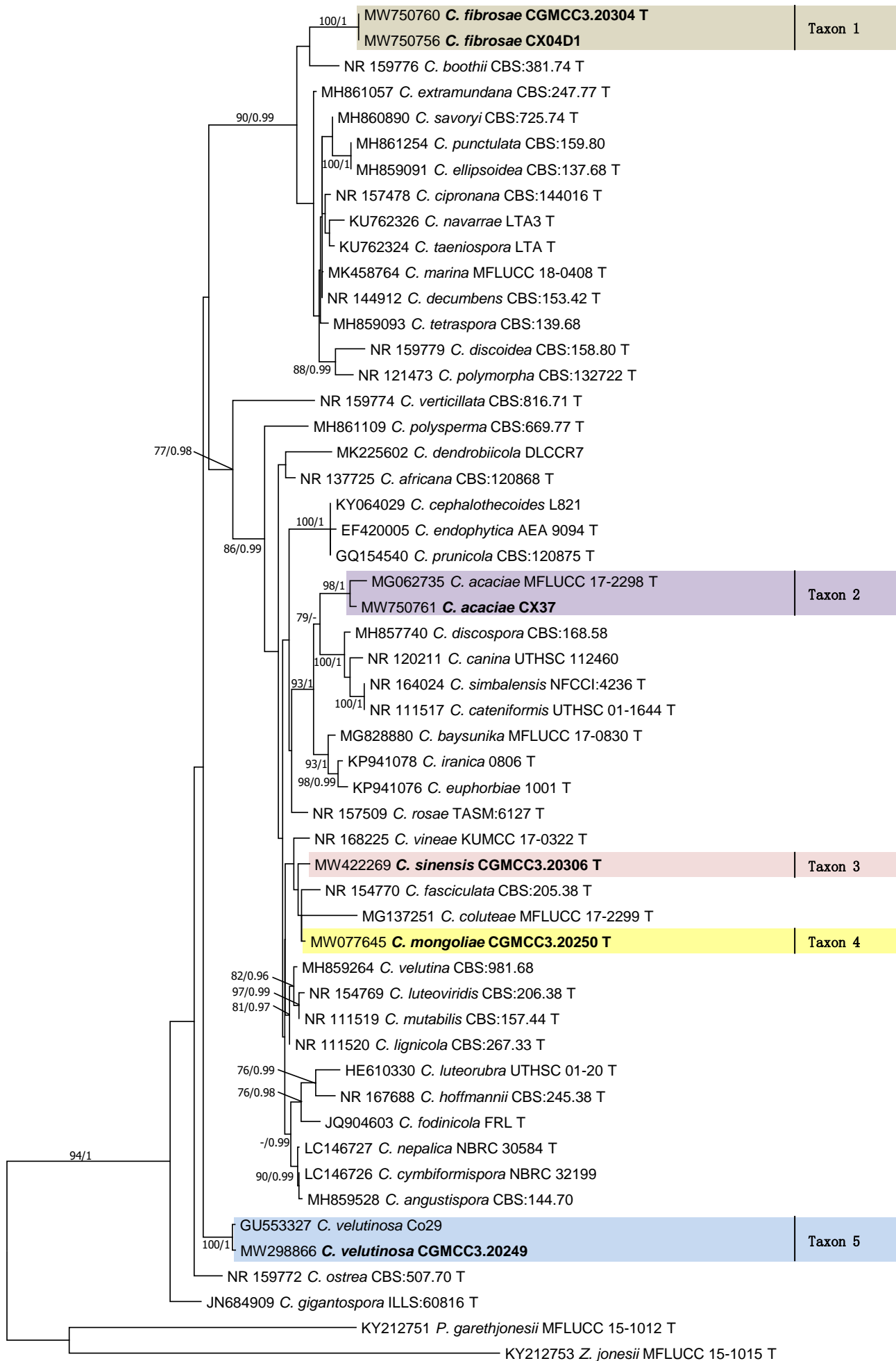

Supplement: Supplementary material 1 — Figure S1. ML tree generated from ITS sequence data [file mycokeys-83-105-s001.pdf]

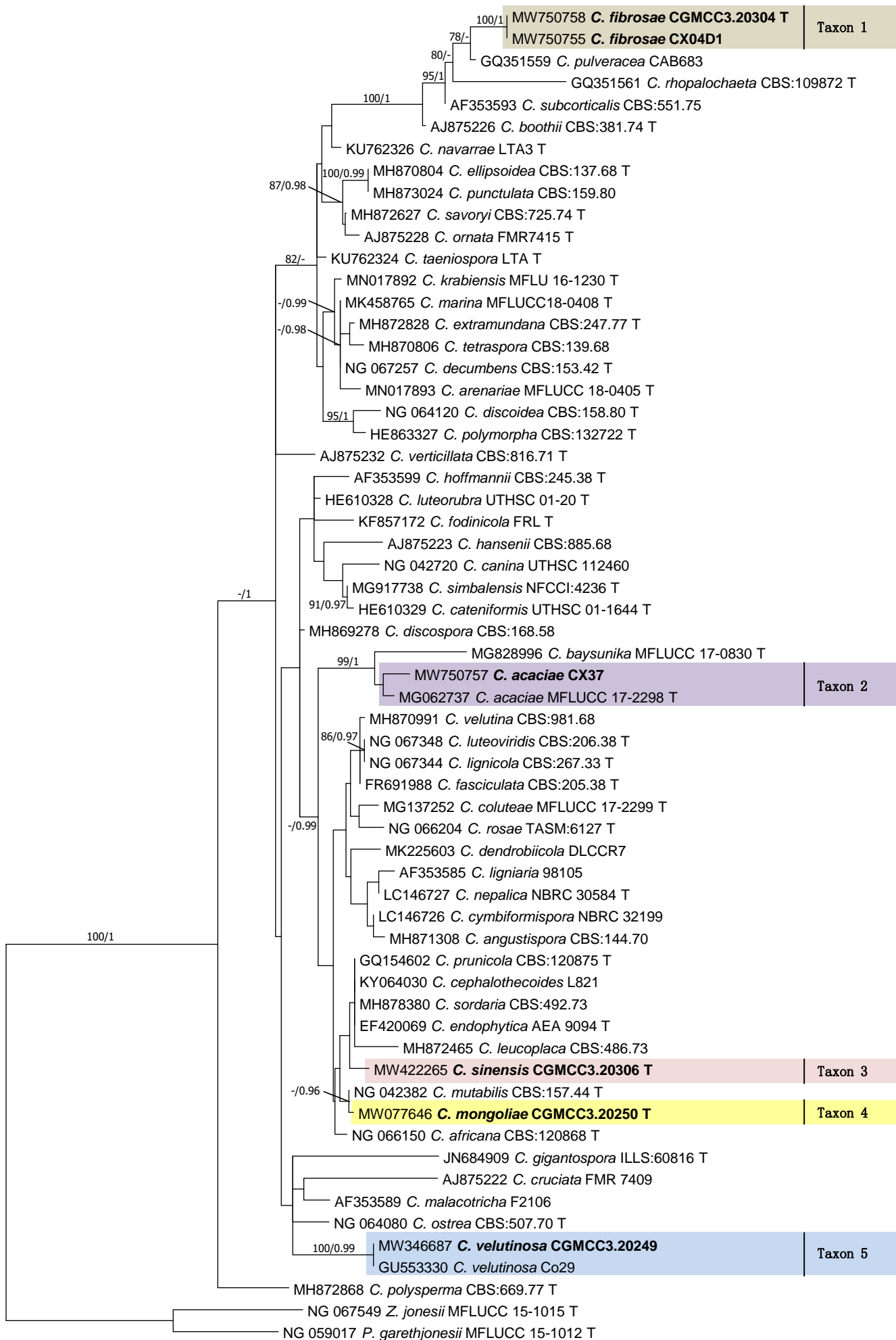

Supplement: Supplementary material 2 — Figure S2. ML tree generated from LSU sequence data [file mycokeys-83-105-s002.pdf]
